# Gross Anatomy and Approach to the Humerus and Femur in the White‐Eared Opossum ( *Didelphis albiventris* )

**DOI:** 10.1111/ahe.70093

**Published:** 2026-02-12

**Authors:** Amanda de Barros Piffer, Layla Contessotto de Oliveira, Letícia Rocha Inamassu, Tais Harumi de Castro Sasahara, Rinaldo José Ortiz, Bruno Cesar Schimming

**Affiliations:** ^1^ São Paulo State University (UNESP), School of Veterinary Medicine and Animal Science, Undergraduate in Veterinary Medicine Botucatu São Paulo Brazil; ^2^ São Paulo State University (UNESP), School of Veterinary Medicine and Animal Science, Graduate Program in Wild Animals Botucatu São Paulo Brazil; ^3^ São Paulo State University (UNESP), Laboratory of Wildlife Anatomy Botucatu São Paulo Brazil

**Keywords:** bones, marsupials, pelvic limb, thoracic limb, wildlife

## Abstract

The white‐eared opossum ( *Didelphis albiventris* ) is a Neotropical marsupial that readily adapts to urban environments. Due to its presence in anthropogenic habitats, this species is frequently exposed to vehicle collisions and dog attacks. Such trauma often results in long bone fractures, with humeral and femoral fractures being common in roadkill specimens. Long bone fractures can be stabilised using external or internal skeletal fixation devices. Therefore, detailed knowledge of the anatomical features of the humerus and femur, as well as the associated muscular and neurovascular structures in the thoracic and pelvic limbs, is essential to guide surgical access to the humeral and femoral shafts in orthopaedic interventions. In this study, eight white‐eared opossums were examined. Based on anatomical dissections and radiographic imaging, the morphology of the humerus and femur, as well as the muscular and nervous anatomy of the thoracic and pelvic limbs, was documented. The observed anatomical structures were largely consistent with those reported for domestic mammals, such as dogs, and wild species, including paca and capybara. Based on these findings, the medial approach is recommended as the most suitable for surgical access to the humeral diaphysis, while the craniolateral approach is most appropriate for access to the femoral diaphysis for the placement of compression plates in the white‐eared opossum.

## 1 Introduction

The family Didelphidae comprises approximately 15 genera and 56 species of Neotropical marsupials, represented primarily by opossums and possums (Cáceres 2012; Rossi et al. 2012). The genus *Didelphis* is composed of six species, most of which are widely distributed. Of these six species, five occur in South America, and four are found in Brazil (Cáceres 2012). Brazilian *Didelphis* include the species *Didelphis albiventris* , *D. aurita* , *D. marsupialis* , and *D. imperfecta* . The white‐eared opossum belongs to the species *D. albiventris* and is one of the most common *Didelphis* in Brazil and it is widely distributed throughout Brazil, occupying several biomes such as Caatinga, Cerrado, Pampa and Atlantic Forest (Gardner 2008; Melo and Sponchiado 2012).

*D. albiventris* are opportunistic and omnivorous animals that have increasingly adapted to urban life. Their omnivorous diet includes fruits, seeds, leaves, insects, molluscs and vertebrates of various sizes. They frequently raid chicken coops and feed on bird eggs and chicks (Flórez‐Oliveros and Vivas‐Serna 2020). This means the white‐eared opossum is frequently observed in both rural and urban environments, and it can be considered one of the Brazilian wild mammal species with the greatest contact with humans. Due to the proximity of the white‐eared opossum to urban areas, the reduction of natural predators and its easy adaptation to urban environments, home invasions, road kills, and attacks by dogs are increasingly common and, consequently, the number of treatments for these traumatised animals in veterinary centres specialised in wild animals has increased.

Typically, vehicle collision causes long bone fractures, which represent one of the main points of orthopaedic treatment in veterinary medicine (Romano et al. 2008). Long bone fractures can be stabilised by external and internal skeletal fixators. Internal fixation can be achieved, for example, through intramedullary pins, bone plates, steel wire cerclages, and locked rods (Carneiro et al. 2017; Fossum 2013; White et al. 2006).

Rodrigues et al. (2009) reported that trauma is common in wild animals; however, there are few reports of surgical procedures in these animals. Leal et al. (2021) cited that humerus and femur fractures are common in roadkill animals. There are descriptions of surgical access in some wild animal species, such as the giant anteater (Sesoko et al. 2016) and paca (Leal et al. 2021), but no studies on surgical access in white‐eared opossums have been observed in specialised literature. The high rates of opossum roadkill on Brazilian highways (Cherem et al. 2007; Miranda et al. 2017; Prada 2004) highlight the importance of anatomical and radiographic studies to establish appropriate treatment for the white‐eared opossum, as anatomical knowledge of the arm and thigh is fundamental to perform osteosynthesis in the humerus and femur. Thus, the objective of this study was to describe the anatomy of the arm and thigh to determine the anatomical basis for surgical access to the diaphysis of the humerus and femur in the white‐eared opossum ( *Didelphis albiventris* ), for placement of compression plates, as these are commonly used for fracture fixation in routine small animal surgery (Field et al. 2018), contributing to future clinical‐surgical studies. Furthermore, this study aimed to fill a gap in the anatomy of these marsupials and contribute information to support veterinary clinical‐surgical care.

## 2 Material and Methods

### 2.1 Animals

Eight white‐eared opossums ( *D. albiventris* ) were used in this study. All specimens originated from the Center of Medicine and Research of Wild Animals (CEMPAS), School of Veterinary Medicine and Animal Science, UNESP, Botucatu, São Paulo, Brazil. Cadavers of animals that died from causes unrelated to this study, and whose thoracic and pelvic limb regions remained unaffected, were collected. The cadavers were subsequently transferred to the Laboratory of Wildlife Anatomy, where they were frozen and later utilised for the investigation of anatomical bases for surgical access to the humerus and femur.

### 2.2 Anatomical and Radiographic Studies

Four opossum specimens were thawed and subsequently fixed in a 10% aqueous formaldehyde solution, in which they remained immersed for 7 days (Pinheiro et al. 2014). Following fixation, the brachial and femoral regions were dissected, and the relevant anatomical structures, including musculature, vascular elements, and peripheral nerves, were identified to establish the anatomical foundations for surgical approaches to the humerus and femur. To refine and adapt these surgical approaches, pertinent literature, such as Johnson (2014) and Leal et al. (2021), was consulted. The anatomical description was therefore elaborated in accordance with the incision lines and access routes recommended for exposure of the humeral and femoral shafts, as outlined by Johnson (2014) and Leal et al. (2021).

Initially, a craniolateral incision was performed on the brachial region, extending proximally from the greater tubercle of the humerus to the lateral epicondyle distally. This approach provided exposure of the humeral midshaft, enabling the dissection and identification of anatomical structures (arteries, veins, nerves, and muscles) located adjacent to the craniolateral surface of the bone. Subsequently, a medial incision was executed, extending from the lesser tubercle proximally to the medial epicondyle distally, with the objective of exposing the entire humeral diaphysis. All arteries, veins, nerves, and muscles encountered within this medial approach were carefully dissected and described.

For access to the femoral shaft, a craniolateral incision was made in the thigh of the white‐eared opossum, extending from the greater trochanter to the femorotibial joint, as recommended by Johnson (2014) and Leal et al. (2021). It is important to note that white‐eared opossums lack a patella in this joint (Inamassu et al. 2017), hence the reference to the femorotibial articulation. This incision allowed exposure of femoral diaphysis, permitting the dissection and identification of arteries, veins, nerves, and muscles associated with the craniolateral aspect of the femur.

The other four opossum carcasses were thawed for radiographic examination at the Diagnostic Imaging Service of the School of Veterinary Medicine and Animal Science, UNESP. Radiographs of the thoracic and pelvic limbs were obtained in mediolateral and craniocaudal projections using a Shimadzu EZY‐RAD radiographic system, with exposure parameters set at 40 kV and 8 mAs.

Following radiographic acquisition, the specimens were subjected to rapid maceration and thermal processing. Soft tissues—including skin and subcutaneous tissues, muscles and associated fasciae, blood vessels, nerves, tendons, and ligaments—were removed to expose the humerus and femur as completely as possible. The cleaned bones were subsequently boiled in water to facilitate the removal of any residual soft tissue, which was manually eliminated using a scalpel and knife. The bones were then bleached by immersion in a 130‐volume hydrogen peroxide solution diluted to 30% for approximately 12 h. After bleaching, they were rinsed under running water to eliminate residual peroxide and dried in sunlight, following the protocol described by Costa (2021). Anatomical structures were identified and described in accordance with the *Nomina Anatomica Veterinaria* (International Committee on Veterinary Gross Anatomical Nomenclature 2017).

## 3 Results

### 3.1 Gross Anatomy of the Humerus and Femur

**FIGURE 1:**
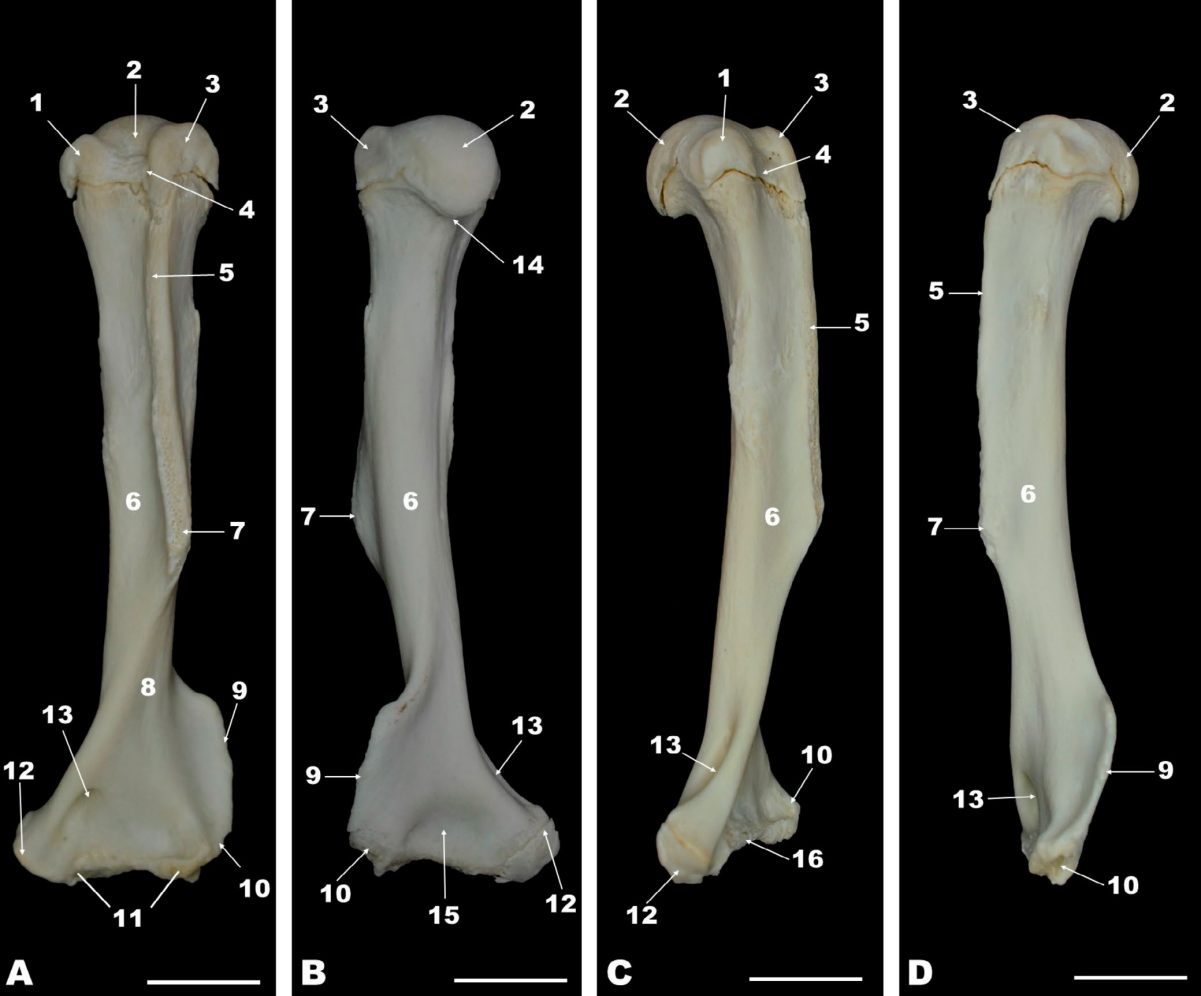
Cranial (A), caudal (B), medial (C), and lateral (D) views of the left humerus of the white‐eared opossum. 1, Lesser tubercle; 2, Humeral head; 3, Greater tubercle; 4, Intertubercular groove; 5, Crest of the greater tubercle; 6, Humeral shaft; 7, Deltoid tuberosity; 8, Brachialis groove; 9, Lateral supracondylar crest; 10, Lateral epicondyle; 11, Humeral condyle; 12, Medial epicondyle; 13, Supratrochlear foramen; 14, Humeral neck; 15, Olecranon fossa; 16, Trochlea. Scale bar = 1 cm.

**FIGURE 2:**
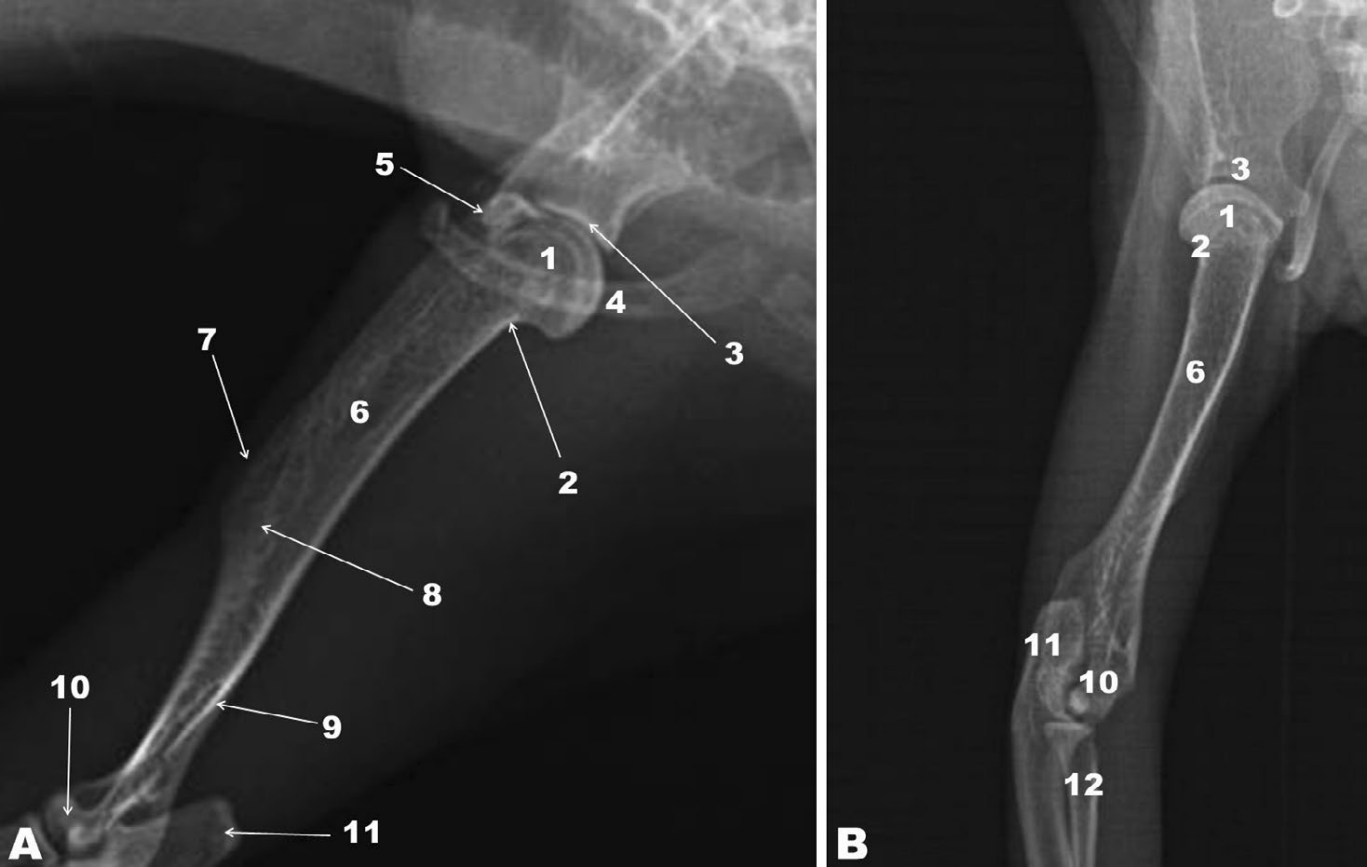
Radiographic image of the humerus of the white‐eared opossum. Mediolateral (A) and craniocaudal (B) projections. 1, Humeral head; 2, Humeral neck; 3, Glenoid cavity of the scapula; 4, Clavicle; 5, Greater tubercle of the humerus; 6, Humeral shaft; 7, Crest of the greater tubercle; 8, Deltoid tuberosity; 9, Lateral supracondylar crest; 10, Humeral condyle; 11, Olecranon tuberosity of the ulna; 12, Radius.

**FIGURE 3:**
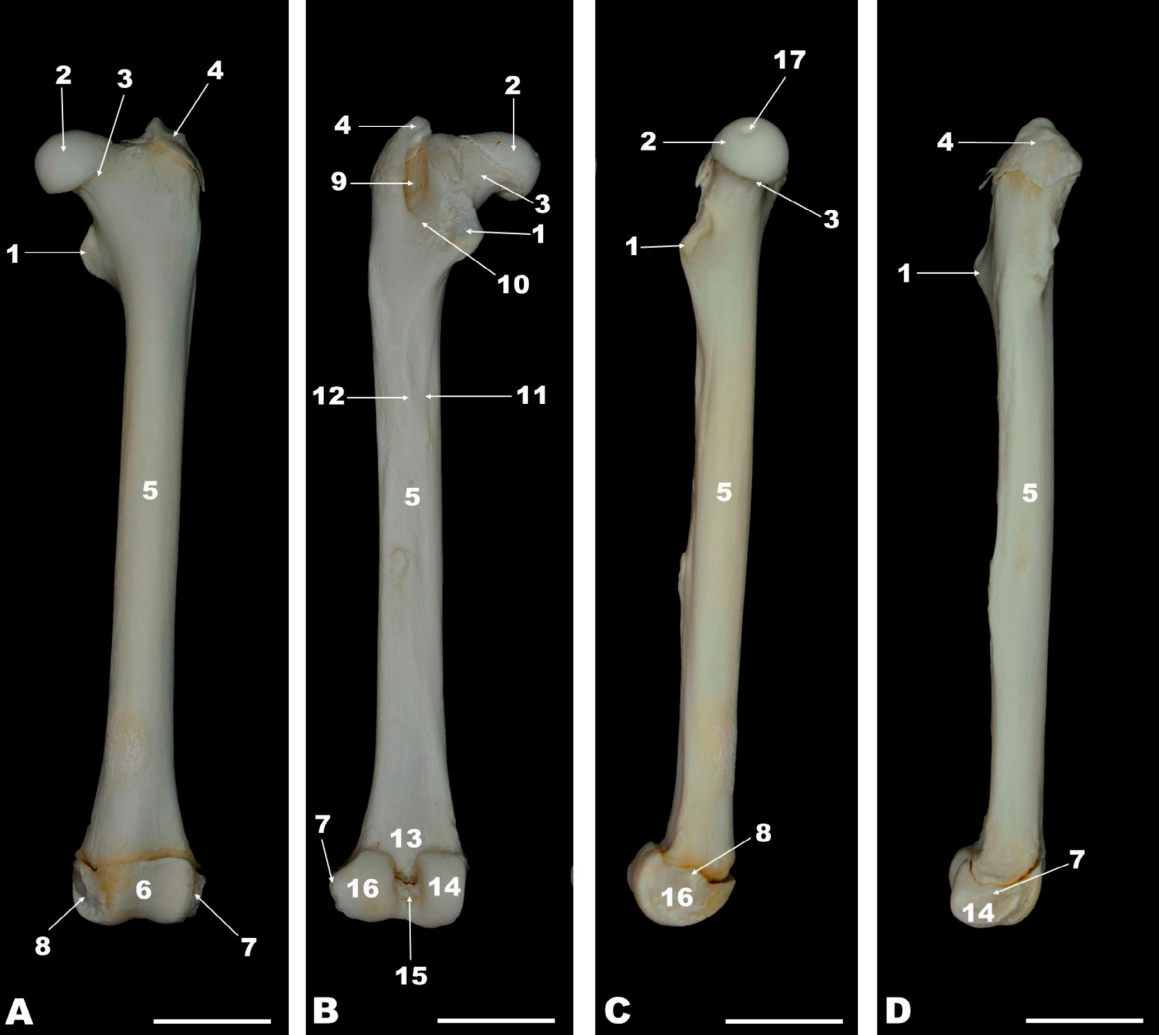
Cranial (A), caudal (B), medial (C), and lateral (D) views of the left femur of the white‐eared opossum. 1, Lesser trochanter; 2, Femoral head; 3, Femoral neck; 4, Greater trochanter; 5, Femoral shaft; 6, Trochlea; 7, Lateral epicondyle; 8, Medial epicondyle; 9, Trochanteric fossa; 10, Intertrochanteric crest; 11, Lateral lip of the *facies aspera*; 12, Medial lip of the *facies aspera*; 13, Popliteal surface; 14, Lateral condyle; 15, Intercondylar fossa; 16, Medial condyle; 17, Fovea of the Femoral head. Scale bar = 1 cm.

**FIGURE 4:**
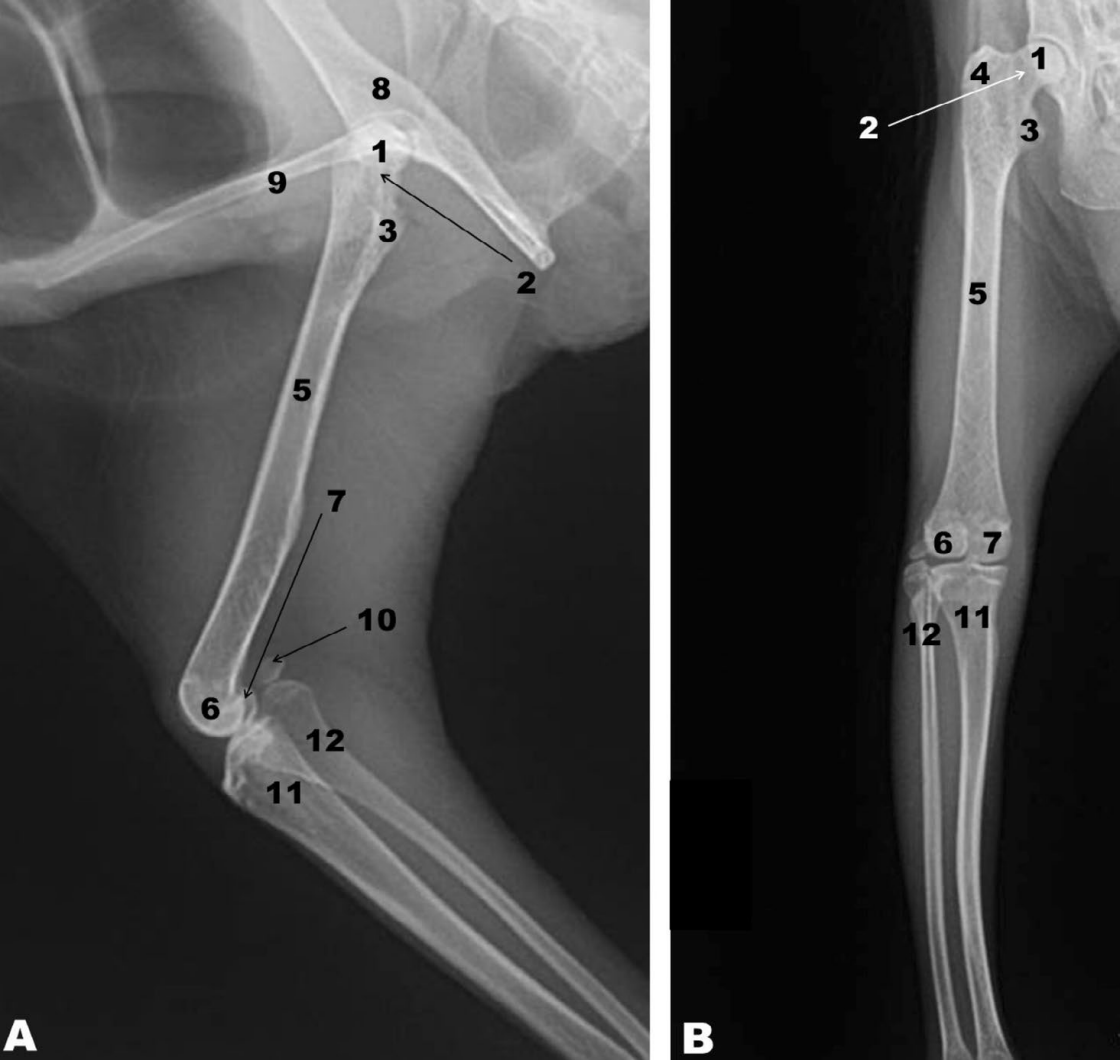
Radiographic image of the femur of the white‐eared opossum. Mediolateral (A) and craniocaudal (B) projections. 1, Femoral head; 2, Femoral neck; 3, Lesser trochanter; 4, Greater trochanter; 5, Femoral shaft; 6, Lateral condyle; 7, Medial condyle; 8, Coxal bone; 9, Epipubic bone; 10, Lateral sesamoid bone of the gastrocnemius muscle; 11, Tibia; 12, Fibula.

The humerus and femur bones found in the arm and thigh regions of the white‐eared opossum were typical long bones, possessing a diaphysis or shaft and two epiphyses, one proximal and one distal (Figures 1, 2, 3, 4). No anatomically relevant variations were identified among the eight opossums examined in this study.

The proximal humeral epiphysis was characterised primarily by the presence of the humeral head and neck, and the greater and lesser tubercles (Figure 1). The humeral head was developed and articulated with the glenoid cavity of the scapula, as observed in the radiographic image (Figure 2). The humeral neck encircled the humeral head. The greater tubercle was craniolateral to the humeral head, and the lesser tubercle was craniomedial. The greater tubercle projected distally through a prominent greater tubercle crest. The intertubercular groove appeared between the greater and lesser tubercles and was unique, as the white‐eared opossum lacked an intermediate tubercle (Figure 1). The greater and lesser tubercles and the crest of the greater tubercle of the humerus were also visualised in the radiographic image (Figure 2). The humeral shaft or body was elongated, and the deltoid tuberosity was located laterally near the distal part of the crest of the greater tubercle of the humerus. The brachialis groove was also observed on the humeral body, near the distal epiphysis. The distal epiphysis of the humerus presented the humeral condyle, which was divided into the trochlea humeri (located medially) and the capitulum humeri (located laterally). The medial and lateral epicondyles of the humerus, the supratrochlear foramen, and the coronoid and radial fossae were also observed. The olecranon fossa was found caudally in the distal epiphysis of the humerus (Figure 1).

The proximal skeleton of the free part of the pelvic limb was formed by the femur. The proximal femoral epiphysis of the white‐eared opossum consisted of a prominent head surrounded by a pronounced neck, and two trochanters (greater and lesser trochanters) (Figure 3). The fovea of the femoral head was found medially. The greater trochanter was lateral to the femoral head, whereas the lesser trochanter was located medially and slightly distal to the femoral head. The trochanteric fossa was located between the greater trochanter and the femoral neck. The intertrochanteric crest was found between the greater and lesser trochanters caudally. The intertrochanteric line was observed on the cranial aspect of the proximal femoral epiphysis (Figure 3). No third trochanter was observed in the femur of the white‐eared opossum. The greater and lesser trochanters of the femur, as well as the head and neck of the femur, were visualised on the radiographic images (Figure 4). The femoral diaphysis was represented by the elongated shaft. The lateral and medial lips of the facies aspera were observed caudally on the femoral shaft, as was the popliteal surface (Figure 3). On the distal epiphysis of the femur, the medial and lateral condyles were observed caudally and a shallow trochlea cranially. The intercondylar fossa was located between the condyles (Figure 3). The medial and lateral condyles of the femur were also visualised on the radiographic images (Figure 4).

Although these structures are not components of the femur and are not located within the thigh region of the white‐eared opossum, it is noteworthy that the coxal and epipubic bones, the lateral sesamoid of the gastrocnemius muscle, as well as the tibia and fibula, were visible in the radiographic images (Figure 4). The absence of the patella in the radiographs is also noteworthy (Figure 4). The epipubic bones are paired structures that articulate with the pubic bones near the pubic symphysis and extend cranially into the abdominal wall, providing support for the abdominal musculature.

### 3.2 Anatomical Basis to the Humerus Shaft

**FIGURE 5:**
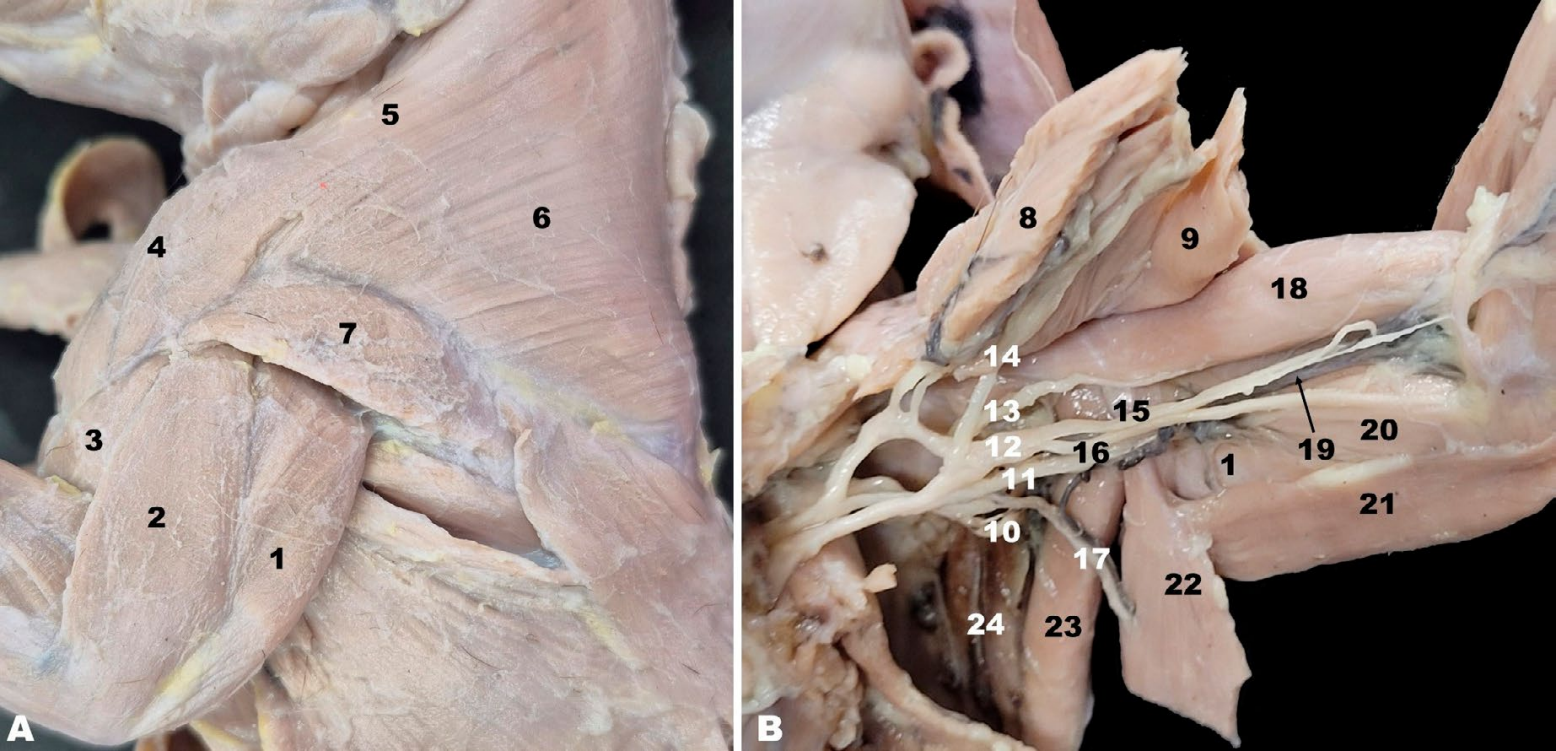
Craniolateral view of the shoulder and brachii regions (A) and medial view of the axilla and brachii regions highlighting the nerves originating from the brachial plexus (B) of the white‐eared opossum. 1, Long head of the triceps brachii muscle; 2, Lateral head of the triceps brachii muscle; 3, Brachialis muscle; 4, Cleidobrachialis muscle; 5, Cleidocephalic muscle; 6, Trapezius muscle; 7, Deltoid muscle; 8, Superficial pectoralis muscle; 9, Deep pectoralis muscles; 10, Axillary nerve; 11, Radial nerve; 12, Common trunk of the median and ulnar nerves; 13, Musculocutaneous nerve; 14, Caudal pectoral nerve; 15, Median nerve; 16, Ulnar nerve; 17, Thoracodorsal nerve; 18, Biceps brachii muscle; 19, Brachial vessels; 20, Medial head of the triceps brachii muscle; 21, Tensor fasciae antebrachii muscle; 22, Latissimus dorsi muscle; 23, Teres major muscle; 24, Subscapularis muscle.

The following muscles were identified on the craniolateral aspect of the white‐eared opossum's brachium: cleidobrachialis, brachialis, deltoid, and the lateral and long heads of the triceps brachii. The cleidobrachialis was a component of the brachiocephalicus muscle, which extended from the brachium to the lateroventral aspect of the neck. From its attachment to the clavicle, the brachiocephalicus was divided into two parts: cleidocephalicus and cleidobrachialis. The latter was a thin segment extending from the clavicle to the humerus, inserting on the distal end of the cranial margin of the humerus, and was located cranially on the brachium (Figure 5). The deltoid muscle in the white‐eared opossum was divided into two parts: the scapular and the acromial parts. The scapular part was the more developed and arose from the spine of the scapula, while the acromial part had a more fusiform appearance and arose from the acromion of the scapula and the clavicle. Both parts inserted on the deltoid tuberosity and the humeral crest (Figure 1). The brachialis occupied the brachialis groove, situated on the lateral aspect of the humeral shaft (Figure 5). It originated from the humeral crest and brachialis groove (Figure 1) and inserted onto the radial and ulnar tuberosities.

The triceps brachii formed a large muscular mass on the lateral side of the brachium and consisted of three heads: lateral, long, and medial. The lateral and medial heads were positioned on the corresponding aspects of the humerus, while the long head, the largest, was primarily located on the caudal aspect but extended partially to the lateral and medial surfaces (Figure 5). The long head originated from the caudal border of the scapula, whereas the lateral and medial heads originated from the humerus. All three heads converged to insert on the olecranon tuberosity of the ulna.

On the medial aspect of the brachium of the white‐eared opossum, the biceps brachii, superficial pectoralis, tensor fasciae antebrachii, and the medial head of the triceps brachii muscles were identified, along with nerves originating from the brachial plexus (Figure 5). The biceps brachii was located along the medial surface of the humerus, originating from the supraglenoid tubercle of the scapula, coursing through the intertubercular groove of the humerus, and inserting onto the radial and ulnar tuberosities. The tensor fasciae antebrachii appeared as a thin muscular sheet overlying the long head of the triceps brachii on the medial aspect of the brachium (Figure 5). This muscle arose from the fascia of the latissimus dorsi and inserted into the forearm fascia at the level of the olecranon of the ulna. The superficial pectoralis inserted on the greater tubercle of the humerus and humeral crest (Figure 1). Its portion near the insertion is of relevance when accessing the medial aspect of the brachium, as the muscle extends laterally from the sternum to the humerus, crossing the medial surface of the arm. The brachial plexus was in the axillary region, with its nerves coursing along the medial aspect of the brachium. The nerves originating from the brachial plexus and innervating these muscles included the axillary, musculocutaneous, cranial pectoral, radial, median, and ulnar nerves (Figure 5). The musculocutaneous, median, ulnar, and radial nerves passed between the caudal border of the biceps brachii and the cranial borders of the medial and long heads of the triceps brachii. Following bifurcation of the common trunk of the median and ulnar nerves, the ulnar nerve extended to the medial epicondyle of the humerus, passing between the humerus and the olecranon. The brachial artery and veins ran alongside the median and ulnar nerves (Figure 5).

### 3.3 Anatomical Basis to the Femoral Shaft

**FIGURE 6:**
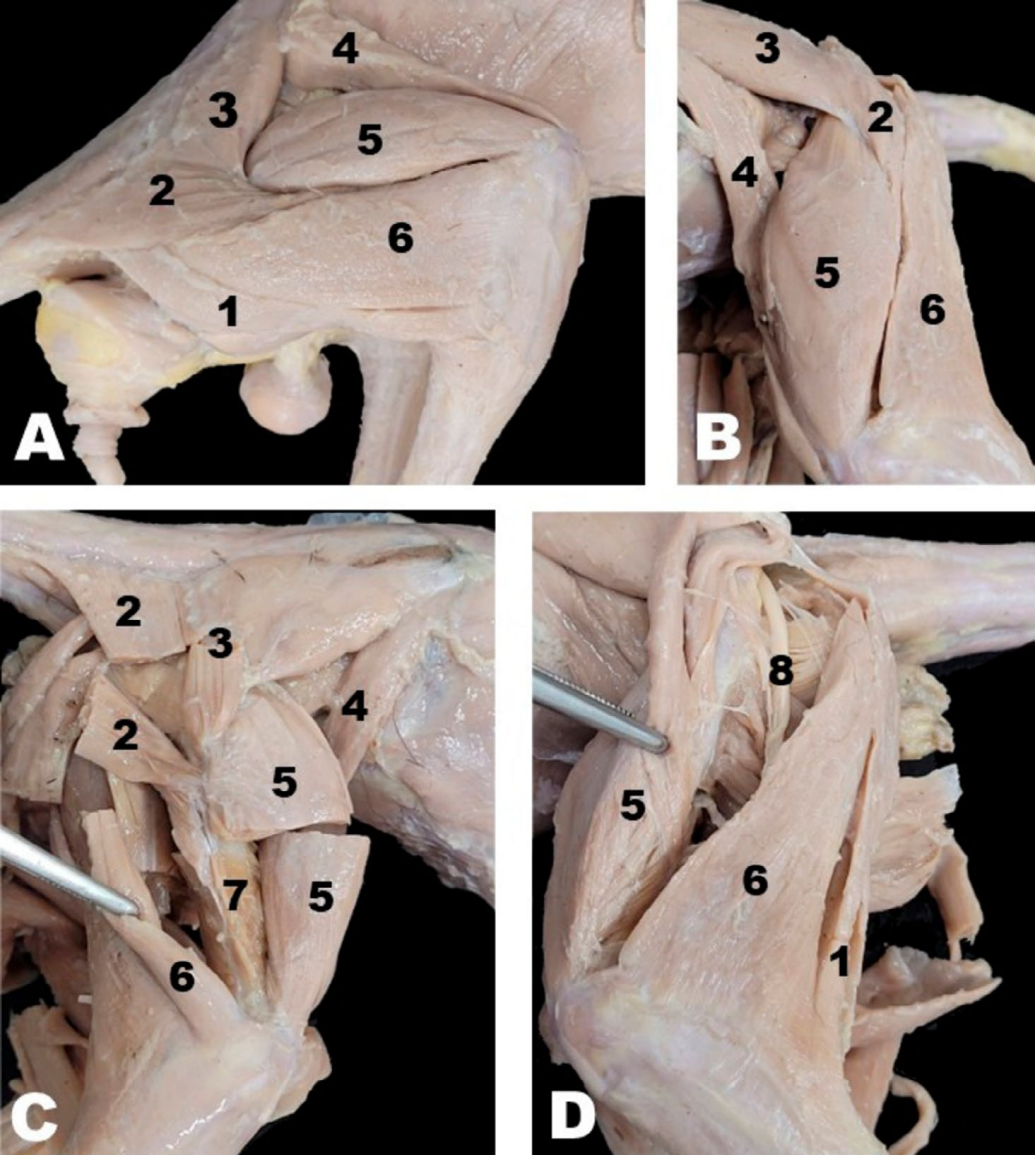
Craniolateral view of the gluteal and femoral regions (A, B) with the superficial muscles sectioned and/or reflected (C, D) of the white‐eared opossum. 1, Semitendinosus muscle; 2, Gluteofemoral muscle; 3, Superficial gluteus muscle; 4, Sartorius muscle; 5, Vastus lateralis muscle; 6, Biceps femoris muscle; 7, Femoral shaft; 8, Sciatic nerve.

On the craniolateral aspect of the white‐eared opossum's thigh, the sartorius, vastus lateralis, rectus femoris, biceps femoris, and gluteofemoris muscles were identified (Figure 6). The sartorius was the most cranially located muscle of this group, consisting of a single belly originating from the coxal tuberosity and lateral margin of the ilium, and inserting onto the proximal epiphysis of the tibia (Figure 6). The vastus lateralis, a head of the quadriceps femoris, was located on the lateral aspect of the femur. The quadriceps femoris muscle was composed of four heads: rectus femoris, vastus lateralis, vastus medialis, and vastus intermedius. The vastus lateralis was covered by the fascia lata; upon incision, a well‐developed fusiform muscle mass extending from the lateral femur to the tibial tuberosity was observed. The rectus femoris, the most cranial head of the quadriceps femoris, was located deep to the sartorius muscle. The biceps femoris was positioned caudolaterally in the thigh, cranial to the semitendinosus and caudal to the vastus lateralis muscles (Figure 6). It extended from the ischial tuberosity to the quadriceps femoris tendon via the fascia lata and to the calcaneus via the common calcanean tendon. The gluteofemoral muscle lay caudal to the superficial gluteus and craniodorsal to the biceps femoris, extending from the first three caudal vertebrae to the lateral aspect of the middle third of the femoral shaft (Figure 6). The sciatic nerve was observed on the lateral aspect of the thigh, deep to the vastus lateralis and biceps femoris muscles. Its location should be considered when accessing the craniolateral aspect of the thigh to expose the femoral shaft (Figure 6).

### 3.4 Surgical Approach to the Humeral Shaft

**FIGURE 7:**
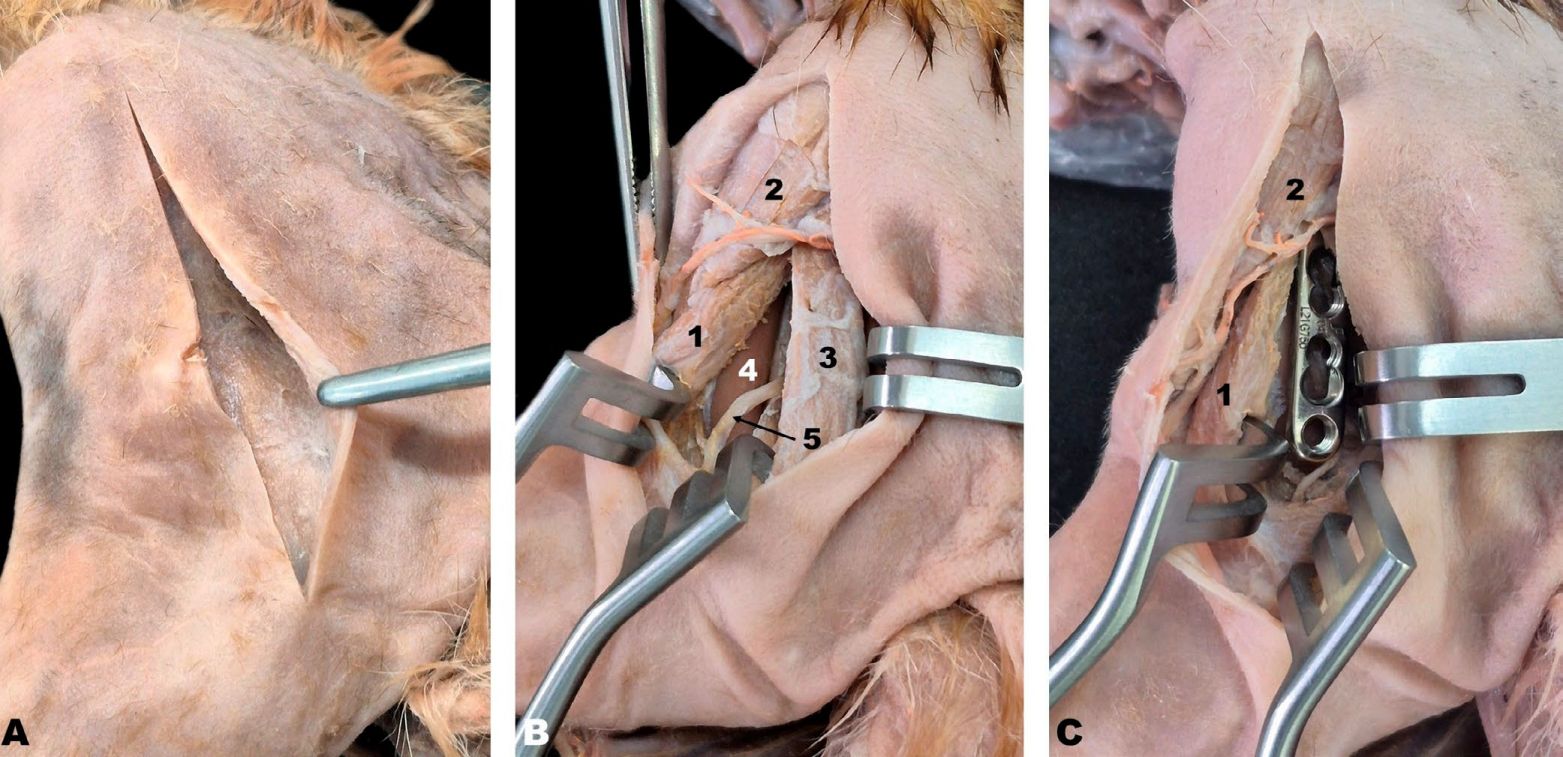
Craniolateral approach to the humeral shaft in the white‐eared opossum (A–C). 1, Brachialis muscle; 2, Deltoid muscle; 3, Lateral head of the triceps brachii muscle; 4, Humeral shaft; 5, Radial nerve.

As initially proposed, craniolateral and medial approaches were performed to access the humeral diaphysis. For the craniolateral approach, the animal was placed in dorsal recumbency. The proximal and distal humeral epiphyses, as well as the humeral crest, were palpated. A longitudinal skin incision was made, extending from the greater tubercle proximally to the lateral epicondyle distally (Figure 7). Following subcutaneous tissue dissection, muscles bound by the muscular fascia were exposed. The cleidobrachialis, deltoid, and triceps brachii muscles (long and lateral heads) were identified and dissected (Figure 7). To access the humeral shaft craniolaterally, the lateral and long heads of the triceps brachii and the brachialis muscle were released and retracted. Notably, the radial nerve was visualised distally following muscle dissection (Figure 7).

**FIGURE 8:**
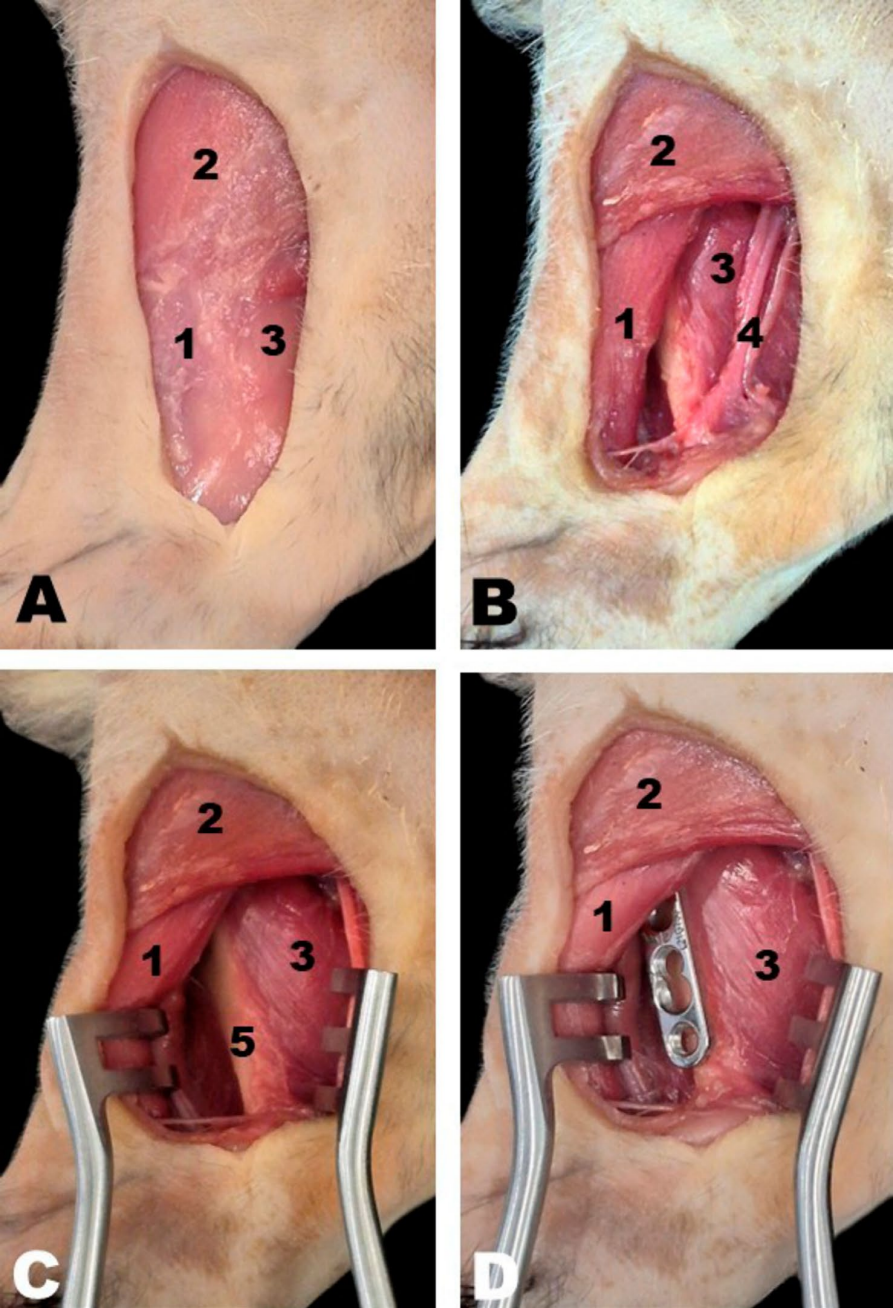
Medial approach to the humeral shaft in the white‐eared opossum (A–D). 1, Biceps brachii muscle; 2, Superficial pectoralis muscle; 3, Medial head of triceps brachii muscle; 4, Brachial vessels and nerves of the brachial plexus; 5, Humeral shaft.

For medial access to the humeral diaphysis, the animal was positioned in dorsal recumbency with the thoracic limbs extended laterally. A longitudinal skin incision was made from the lesser tubercle proximally to the medial epicondyle distally (Figure 8). Dissection of the subcutaneous tissue exposed the superficial pectoralis and biceps brachii muscles, which were identified and retracted. Brachial plexus nerves and brachial vessels were observed caudal to the biceps brachii. To expose the humeral shaft medially, the biceps brachii was displaced cranially, while the brachial plexus nerves, brachial vessels, and medial head of the triceps brachii were retracted caudally, allowing full visualisation of the humeral shaft (Figure 8).

### 3.5 Surgical Approach to the Femoral Shaft

**FIGURE 9:**
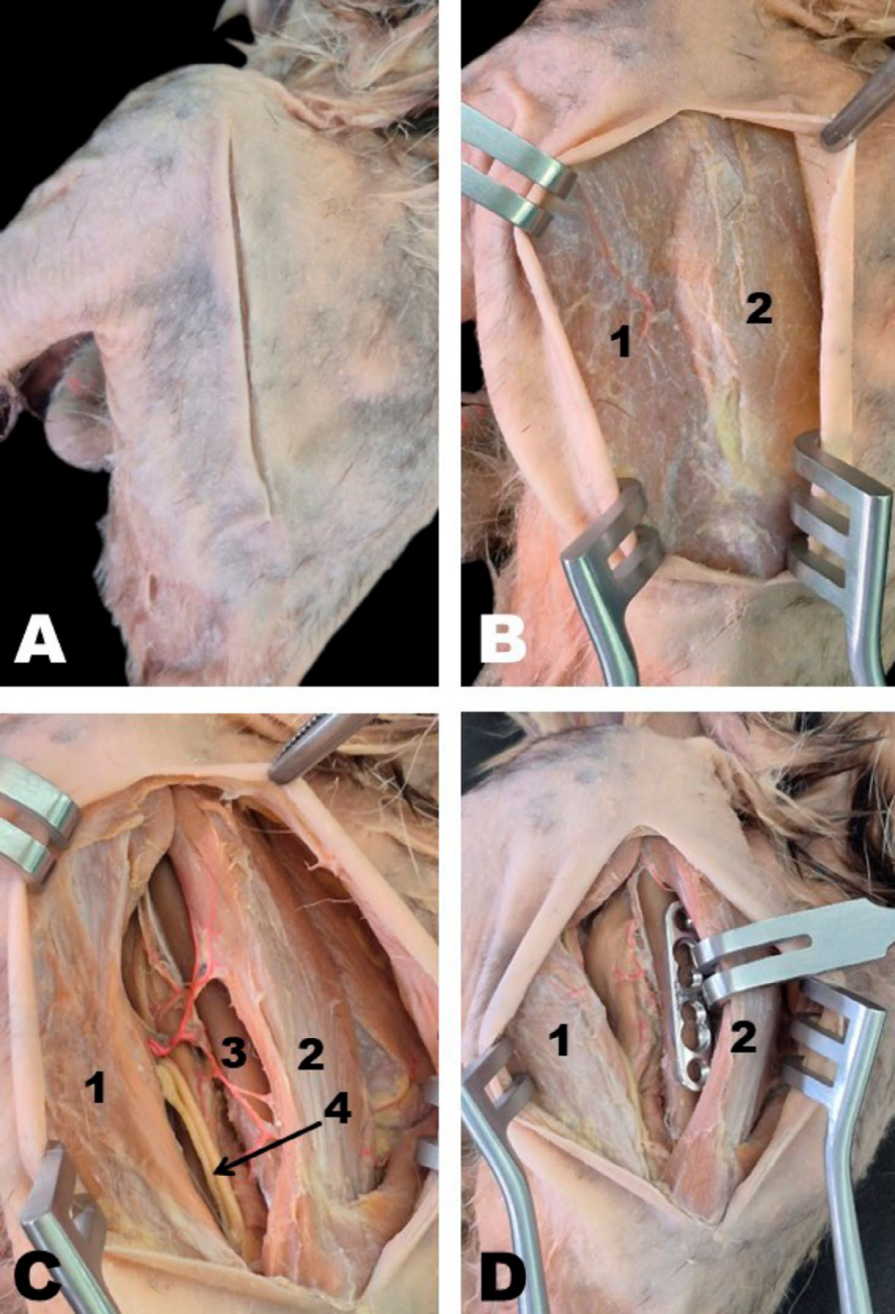
Craniolateral approach to the femoral shaft in the white‐eared opossum (A–D). Skin incision in (A); Dissection of the subcutaneous tissue and muscular fascia with exposure of the muscles in (B). 1, Vastus lateralis muscle; 2, Biceps femoris muscle; 3, Femoral shaft; 4, Sciatic nerve.

The craniolateral approach provided access to the femoral shaft. Anatomical landmarks were established by a longitudinal skin incision extending from the greater trochanter to the femorotibial joint, as the patella is absent in this species and cannot serve as a reference point (Figure 9). Dissection of the subcutaneous tissue exposed the biceps femoris and vastus lateralis muscles, as well as the fascia lata (Figure 9). Subsequent dissection of the biceps femoris and vastus medialis muscles allowed visualisation of the lateral femoral shaft. Further separation of the vastus lateralis muscle enabled exposure of the craniolateral aspect of the femur (Figure 9).

## 4 Discussion

The present study described the anatomical features of the humerus and femur, the anatomical base to the humeral and femoral shaft, and suggests a surgical approach to the humeral and femoral shaft in the white‐eared opossum ( *D. albiventris* ) using gross anatomy and radiographic images with the aim to provide anatomical knowledge for the surgical procedures with these bones in this Neotropical marsupial.

Based on the anatomical and radiographic images, the anatomical characteristics of the humerus, femur, and surrounding soft tissues of the white‐eared opossum could be clearly identified. The humeral structures observed did not differ substantially from those described for domestic mammals such as dogs (Hermanson et al. 2019). Among the most notable features are the well‐developed humeral crest and the supratrochlear foramen. In a previous study on *D. albiventris* , Chiarello et al. (2021) indicated that the humerus of this species exhibits a pronounced humeral crest, which serves as the insertion site for fibres of the pectoral musculature. A strongly developed pectoral muscle is likely associated with an enhanced capacity for adduction at the scapulohumeral joint, a functional attribute that may be essential for the arboreal and fossorial behaviours exhibited by these animals.

The supratrochlear foramen located distally in the humerus of the white‐eared opossum has also been described in domestic dogs (Hermanson et al. 2019). According to these authors, no anatomical structures traverse this foramen, and it may be absent in individuals with very small humeri. However, other reports indicate that the presence of this foramen is associated with a distinct course of the median nerve and brachial artery in species in which it occurs (Chiarello et al. 2021; Kardong 2017). No anatomical structure passed through the supratrochlear foramen in the opossums studied, similar to the dogs and unlike felines. Consequently, this foramen should be considered the distal limit for orthopaedic implant placement, as critical neurovascular structures pass in close proximity to it (Magalhães et al. 2023).

The anatomical features of the femur observed in the white‐eared opossum in this study were consistent with those reported for other wild mammals, including the marsh deer (Schimming et al. 2015), paca and capybara (Araújo et al. 2013; Silveira and Assis Neto 2024), as well domestic dogs (Hermanson et al. 2019). Radiographic analysis also enabled the identification of the epipubic bone, whereas the patella was not visualised. Epipubic bones are characteristic of marsupials (Guillon et al. 2021). The absence of an osseous patella in the white‐eared opossum has been previously documented by Inamassu et al. (2017) and is considered a typical marsupial anatomical trait. This absence may also account for the shallow trochlear groove observed in the distal femoral epiphysis of the specimens examined in this study.

The humeral shaft of the white‐eared opossum was accessed through craniolateral and medial approaches. A principal challenge associated with the craniolateral approach was the presence of tightly adherent musculature, which impeded effective separation along the muscle fascia. Additionally, the radial nerve traverses transversely across the humeral shaft, which may complicate surgical exposure and the placement of compression plates in this region. The pronounced development of the deltoid tuberosity further contributes to the difficulty of plate application via the craniolateral route. Similar observations were reported by Leal et al. (2021), who noted that craniolateral access to the humerus in the paca is challenging due to the bone's anatomical features. Magalhães et al. (2023) also indicated that both medial and lateral aspects of the humerus can be used for exposure of the humeral shaft; however, they emphasised that the lateral approach is more complex and requires extensive muscular detachment.

The presence of the brachial plexus on the medial aspect of the arm in the white‐eared opossum may render medial surgical access to the humerus challenging, as this region contains numerous neurovascular structures that require careful manipulation. Nevertheless, by cranially displacing the biceps brachii muscle and caudally the medial head of the triceps brachii and the nerves and blood vessels, it was possible to expose the humeral shaft, since these structures shift concomitantly with the muscle. Leal et al. (2021) noted that medial surgical access to the humeral shaft is advantageous because the medial surface of the humerus is nearly flat, facilitating the placement of orthopaedic plates. The present findings corroborate this observation. Furthermore, the medial approach is widely employed because fewer muscle groups occupy this region, allowing the diaphysis to be accessed simply by cranially reflecting the biceps brachii muscle (König and Liebich 2021; Magalhães et al. 2023).

However, the position of the biceps brachii on the medial aspect of the arm can also pose a disadvantage during medial exposure of the humeral shaft. To overcome this limitation, the muscle must be displaced either cranially or caudally, as described by Piermattei et al. (2009). Our findings support this recommendation, as the biceps brachii was displaced cranially during the simulated medial approach. This manoeuvre was selected because it allowed simultaneous caudal displacement of the nerves arising from the brachial plexus, thereby providing safer and more efficient access to the humeral shaft in the white‐eared opossum.

In the craniolateral approach to the femur, the biceps femoris and vastus lateralis muscles were found to be firmly adherent to the femoral shaft, necessitating more extensive dissection to adequately expose the bone. Additionally, a more pronounced cranial retraction of the vastus lateralis muscle is recommended to access the craniolateral surface of the femur, as this region is comparatively flatter and therefore more suitable for the placement of compression plates. These findings corroborate with those reported by Leal et al. (2021) for the paca, in which the authors indicated that the lateral aspect of the thigh provides the most effective surgical access to the femoral shaft. In that species, separation of the biceps femoris and vastus lateralis muscles was likewise required to permit exposure of femoral diaphysis.

In conclusion, craniolateral and medial approaches to the humeral shaft, as well as a craniolateral approach to the femoral shaft in the white‐eared opossum, were described with the objective of determining the most suitable surgical access routes to these bones. Sequential dissections, in conjunction with an extensive literature review, enabled accurate anatomical identification. Species‐specific anatomical knowledge proved essential for the recognition of key structures involved in these procedures. The anatomical basis for the approaches described here were consistent with those reported for dogs (Fossum 2013; Johnson 2014; Piermattei et al. 2009), giant anteater (Magalhães et al. 2023; Sesoko et al. 2016), and paca (Leal et al. 2021). Based on our findings, we suggest that the medial approach is the most appropriate for surgical access to the humeral diaphysis, whereas the craniolateral approach provides the most advantageous access to the femoral diaphysis for the placement of compression plates in the white‐eared opossum.

## Conflicts of Interest

The authors declare no conflicts of interest.

## Data Availability

The data that support the findings of this study are available on request from the corresponding author. The data are not publicly available due to privacy or ethical restrictions.
